# Perinatal Lead (Pb) Exposure Results in Sex-Specific Effects on Food Intake, Fat, Weight, and Insulin Response across the Murine Life-Course

**DOI:** 10.1371/journal.pone.0104273

**Published:** 2014-08-08

**Authors:** Christopher Faulk, Amanda Barks, Brisa N. Sánchez, Zhenzhen Zhang, Olivia S. Anderson, Karen E. Peterson, Dana C. Dolinoy

**Affiliations:** 1 Department of Environmental Health Sciences, University of Michigan School of Public Health, Ann Arbor, Michigan, United States of America; 2 Department of Biostatistics, University of Michigan School of Public Health, Ann Arbor, Michigan, United States of America; 3 Center for Human Growth and Development, University of Michigan, Ann Arbor, Michigan, United States of America; 4 Department of Nutrition, Harvard School of Public Health, Boston, Massachusetts, United States of America; University of Santiago de Compostela School of Medicine - CIMUS, Spain

## Abstract

Developmental lead (Pb) exposure has been associated with lower body weight in human infants and late onset obesity in mice. We determined the association of perinatal Pb exposure in mice with changes in obesity-related phenotypes into adulthood. Mice underwent exposure via maternal drinking water supplemented with 0 (control), 2.1 (low), 16 (medium), or 32 (high) ppm Pb-acetate two weeks prior to mating through lactation. Offspring were phenotyped at ages 3, 6, and 9 months for energy expenditure, spontaneous activity, food intake, body weight, body composition, and at age 10 months for glucose tolerance. Data analyses were stratified by sex and adjusted for litter effects. Exposed females and males exhibited increased energy expenditure as compared to controls (p<0.0001 for both). In females, horizontal activity differed significantly from controls (p = 0.02) over the life-course. Overall, food intake increased in exposed females and males (p<0.0008 and p<0.0001, respectively) with significant linear trends at 9 months in females (p = 0.01) and 6 months in males (p<0.01). Body weight was significantly increased in males at the medium and high exposures (p = 0.001 and p = 0.006). Total body fat differed among exposed females and males (p<0.0001 and p<0.0001, respectively). Insulin response was significantly increased in medium exposure males (p<0.05). Perinatal Pb exposure at blood lead levels between 4.1 µg/dL and 32 µg/dL is associated with increased food intake, body weight, total body fat, energy expenditure, activity, and insulin response in mice. Physiological effects of developmental Pb exposure persist and vary according to sex and age.

## Introduction

The developmental origins of health and disease (DOHaD) hypothesis posits that early life conditions and exposures, particularly during sensitive windows of development, can have long-term health effects [1], [2]. Developmental exposure to the heavy metal lead (Pb) has been associated with decreased fetal and childhood growth, and risk of delayed puberty in humans [3]–[7] and early onset of puberty, and late onset obesity in rodents [8], [9]. Human studies of the association of Pb exposure with weight status are conflicting. Bone measures of chronic Pb exposure have been both positively and negatively correlated with BMI at different ages, but findings may be related to differences in cross-sectional and longitudinal study designs [10], [11].

Developing fetuses are particularly vulnerable to Pb exposure, as both transplacental and lactational transfer from mother to fetus are more efficient than oral ingestion [12]. Additionally, Pb can persist in the human body and increased bone turnover during pregnancy and lactation causes Pb stored in the bones to be mobilized into the bloodstream further increasing the Pb burden on the fetus [13]. Though the half-life of Pb depletion in blood is around 30 days in humans, the initial rise to peak levels in rodents occurs much sooner. For example, mice exposed to Pb-acetate (10 and 25 mg/kg) in a pulsed dose experiment showed a peak blood lead level (BLL) (55–120 µg/dL) at 24 hours, followed by a rapid decline to less than 10 µg/dL by 20 days at which time, mice continue to perform poorly in standard maze tests [14]. While some outcomes of early-life Pb exposure such as altered neurodevelopment are well characterized, others are not. For example, few human or animal studies have attempted a life-course approach to investigate the effects of early-life Pb exposure on the metabolism and body habitus. In human cohort studies of prenatal Pb exposure, weight and body mass index (BMI) have followed subjects into early childhood [6], [15]. One recent study of Pb exposure in mice followed a longitudinal design, although the characterization of metabolic effects was restricted to body weight at 1 year of life [9].

Due to its historically widespread use in gasoline and paint, and its persistence in the environment, Pb remains a health threat in the United States and around the world [16]. Major legislation has limited Pb use in gasoline and paint in the United States, resulting in a decline in average BLL from 12.8 µg/dL to 2.8 µg/dL between 1976 and 1991, and a further decrease to 1.64 µg/dL by 2002 [17]–[19]. Despite regulations, Pb persists in the environment in older homes and buildings, soil, dust, food and water, leading to exposure most commonly by ingestion or inhalation. As of yet, no safe level of Pb exposure has been established and adverse health effects are increasingly being found at lower levels of exposure [20], [21].

In the present study, we assess the effects of prenatal and early-life exposure to multiple physiologically relevant levels of Pb on energy expenditure, spontaneous motor activity, food intake, weight, total body fat, glucose tolerance and insulin response, by performing metabolic phenotyping at multiple time-points into adulthood. A perinatal rather than life-course exposure model was chosen to model relevant Pb exposure during the most sensitive developmental milestones of gestation and infancy and to isolate the effects of perinatal exposure on later-in-life metabolic shifts.

## Methods

### Animals and exposure

All mating mice were obtained from a colony of *A^vy^* Agouti strain mice maintained for over 220 generations with forced heterozygosity for the *A^vy^* allele through the male line, resulting in a genetically invariant background with 93% similarity to C57BL/6J [22], [23]. To minimize the effects of age and parity, virgin *a/a* dams were randomly assigned to one of four Pb treatment groups. Following 2 weeks on their respective Pb-acetate water, virgin *a/a* females (6–8 weeks of age) were mated with *A^vy^/a* males (7–10 weeks of age). Exposure was via drinking water supplemented with Pb-acetate at (a) 0 ppm (n = 11 litters, control), (b) 2.1 ppm (n = 12 litters, low), (c) 16 ppm (n = 12 litters, medium), or (d) 32 ppm (n = 14 litters, high). Leaded water was made by dissolving lead(II) acetate trihydrate (Sigma-Aldrich) in single batches of distilled water. Pb in water concentrations above the limit of detection (LOD), 1.3 µg/dL, were verified using inductively coupled plasma mass spectrometry (ICPMS; NSF International, Ann Arbor, MI). Exposure was maintained throughout gestation and lactation. BLL was measured in dams at weaning after approximately 8 weeks of exposure.

Mating *a/a* females with *A^vy^/a* males resulted in litters of approximately 50% *a/a* and 50% *A^vy^/a* pups. To avoid known confounding factors, only genotype *a/a* offspring were used in this study. After weaning, pups were weighed and switched to untreated drinking water.

Animals were maintained on a phytoestrogen-free AIN-93G diet (TD.95092, 7% Corn Oil Diet, Harlan Teklad, Madison, WI) throughout the duration of the experiment. All animals had *ad libitum* access to food and drinking water throughout the experiment, while housed in polycarbonate-free cages, and were maintained in accordance with the Institute of Laboratory Animal Resources guidelines [24]. Animals were treated humanely and with regard for alleviation of suffering. The study protocol was approved by the University of Michigan Committee on Use and Care of Animals.

### Life-course evaluation

Only *a/a* offspring were tracked into adulthood for life-course phenotyping. *A^vy^/a* pups were not phenotyped, to avoid confounding by epigenetically programmed obesity [25]. However, *a/a* mice were housed with same-sex *A^vy^/a* siblings to avoid psychological disturbances between phenotyping time points. Designated *a/a* mice were weighed weekly. At 3, 6, and 9 months of age they were transferred to the Animal Phenotyping Core at the Michigan Nutrition Obesity Research Center (MNORC; University of Michigan, Ann Arbor, MI) for complete profiling of energy expenditure, food intake, spontaneous activity, weight, and body composition, as previously described [26] (Methods S1).

During each visit, energy expenditure, spontaneous activity, and food intake were measured continuously for the monitoring period, but were summarized (averaged) into three 12-hour averages during light periods, and 12-hour averages during dark periods. Thus, for each of the three visits to the MNORC, six measures were available for each mouse. Following the 72-hour period of CLAMS (Comprehensive Lab Animal Monitoring System) at the 9-month visit to the MNORC, animals were fasted for 5 hours prior to administration of an oral glucose tolerance test (GTT) by oral gavage, with a glucose dose of 2 g/kg, as previously described [26] (Methods S1). For GTT, a subset of 109 animals were used. At 10 months of age, all animals were euthanized via CO_2_ asphyxiation followed by cervical dislocation. Females were euthanized on the first day of estrus according to vaginal cytology.

### Data analysis

Given that mice are nested in litters, the general approach to estimate exposure-outcome associations was to use linear mixed models that included random intercepts for litter to account for within litter correlation (i.e., similarity of siblings). Since repeated measures for each mouse were taken (e.g., over the life course), the models also accounted for correlation among repeated observations within mice using autoregressive correlation structures unless noted below. For all outcomes we conducted ‘overall sex by exposure’ tests of whether the association between exposure and the life course pattern of the outcome differed by sex, i.e., sex by exposure by age (3, 6, or 9 months) interactions. When these interactions were significant or when strong sex differences in the outcome were present, we continued analyses with similar types of models stratifying by sex. Sex-specific analyses have also been recommended by NIH’s Office of Research on Women’s Health [27]. In sex-stratified models we first examined whether the life course pattern of the outcome differed by exposure group (i.e., age by exposure interaction), and reported this as ‘overall tests’. When these overall tests were significant, we then assessed the exposure differences at each time of measurement. Because nonlinear dose response associations and nonlinear life course patterns have been observed for these outcomes, both age and exposure group were modeled using indicator variables.

The approach described above was applied to all outcomes with modifications for specific outcomes as follows. Models for energy expenditure, spontaneous activity, and food intake were additionally adjusted for light cycle by including a predictor variable that indicated if the measure corresponded to a light or dark cycle. Data analysis on weekly weight measurements used week of measurement as the age variable (instead of age = 3, 6, 9 months) coded as indicator variables. For interpretation purposes, we calculated differences in weight by exposure within weeks 1–9, 12–22, and 24–35, which corresponded to weeks when all mice were at the colony housing location. Measures of glucose and insulin were available only at 9 months, but similar mixed models were used since repeated measures were taken after glucose administration in each mouse. Repeated glucose and insulin measures were also summarized as the area under the curve (AUC), and we examined exposure differences in the AUC. Homeostatic model assessment of insulin resistance (HOMA-IR) was calculated for each animal using baseline glucose and insulin levels [28]. Within the mixed models, linear trend analyses were performed to determine significance in linear trends between lead exposure and body fat or average food intake. Standard errors are designated by the ± symbol and the complete table of standard errors for the measurements above are listed in File S1.

## Results

**Figure 1:**
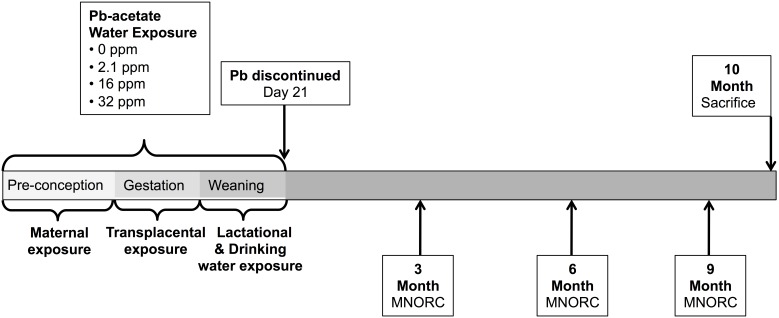
Lead Exposure Timeline. Dams were exposed 2 weeks prior to mating and throughout pregnancy. Pups continued exposure post-natally during lactation and via direct exposure in drinking water until weaning at day 22. After discontinuation of Pb exposure, mice were assayed for physiological parameters at 3, 6, and 9 months of age at the Michigan Nutrition Obesity Research Center (MNORC), and sacrificed at 10 months of age.

The exposure paradigm consisted of experimentally verified Pb-acetate treated distilled water given *ad libitum* to dams through weaning (Fig. 1). Mean maternal BLL, tested at weaning, were below the LOD for the control group, and 4.1 (±1.3) µg/dL, 25.1 (±7.3) µg/dL, and 32.1 (±11.4) µg/dL in the three exposure groups, 2.1 ppm, 16 ppm, and 32 ppm, respectively [29]. Average litter size at birth was 7.04 offspring per dam. Although compared to controls, pup survival in the 2.1 ppm group was reduced by 14% at the time of weaning (due to poor maternal care in two litters), there were also no significant differences in litter size at weaning [29]. At weaning there were, on average, 6.41 offspring per dam: control group (n = 11 litters, 78 offspring, with an average of 7.09±0.16 pups per litter); 2.1 ppm group (n = 12 litters, 68 offspring, with an average of 5.6±0.18 pups per litter); 16 ppm group (n = 12 litters, 86 offspring, with an average of 7.17±0.15 pups per litter); 32 ppm group (n = 14 litters, 75 offspring, with an average of 5.35±0.17 pups per litter) [29]. Longitudinal phenotypic measures were taken from a total of 120 *a/a* mice, on average 2.7 mice per litter (range 1–6 mice from each litter): control (n = 11 litters, 30 offspring); 2.1 ppm group (n = 12 litters, 28 offspring); 16 ppm group (n = 12 litters, 33 offspring); 32 ppm group (n = 14 litters, 29 offspring).

### Overall tests

The association between exposure and the life course pattern of all phenotypes differed significantly by sex at p<0.0001, except for: food intake (p = 0.012), fat mass (p = 0.02), fluid mass (p = 0.01), activity (p>0.2 for all activity types), and hormones (glucose, p = 0.09; insulin, p = 0.99; HOMA-IR, p = 0.07). However, there were marked sex differences in average activity and hormone levels. Therefore, below we report on exposure differences in life course patterns (overall age by exposure tests and differences at specific ages) from sex-stratified analyses.

### Energy expenditure

**Figure 2:**
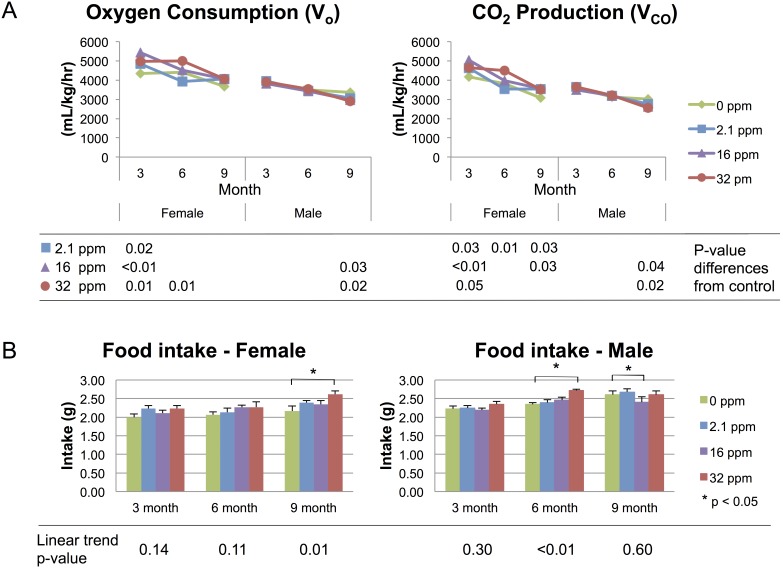
Energy Expenditure and Food Intake. Energy expenditure (mL/kg/hr) measured over a 72-hr period by indirect calorimetry. (A) Oxygen consumption and carbon dioxide (CO_2_) production decline with age, across both sexes and all exposure groups. P-values represent differences from control. (B) Food intake across the life-course, stratified by sex. Stars indicate p-values<0.05 as compared to control. Linear trend p-values represent exposure-dependent trend significance.

Both sexes showed an age-dependent decline in oxygen consumption (V_O_) and carbon dioxide (CO_2_) production (V_CO_); although females consistently had higher levels of both (Fig. 2a, 2b). Female V_O_ ranged from 4291–5374 mL/kg/hr at 3 months, 3921–4924 mL/kg/hr at 6 months, and 3619–4051 mL/kg/hr at 9 months, whereas the ranges for males were 3796–3919 mL/kg/hr at 3 months, 3413–3527 mL/kg/hr at 6 months, and 2889–3372 mL/kg/hr at 9 months (Fig. 2a). Mean V_O_ levels during the life course differed across exposure groups for females and males (p<0.0001 and p<0.0001, respectively). In females, V_O_ among all exposed groups was at least 500 mL/kg/hr higher relative to controls at the 3-month time point (p = 0.02, p<0.01, p = 0.01 for 2.1 ppm, 16 ppm, and 32 ppm, respectively), but by 6 months of age, only the 32 ppm exposure group had significantly higher V_O_ relative to control (p = 0.01). By 9 months of age V_O_ differences across exposure groups were no longer significant. In contrast, males did not show exposure dependent V_O_ differences until the 9-month time point: the 16 ppm and 32 ppm groups had higher V_O_ relative to control (p = 0.03, p = 0.02, respectively) (Fig. 2a) with the largest of these differences approximately 400 mL/kg/hr.

Mirroring the V_O_ findings, female V_CO_ was higher than among males (ranged from 4102–4980 mL/kg/hr at 3 months, 3535–4400 mL/kg/hr at 6 months, and 3012–3538 mL/kg/hr at 9 months in females, ranges for males were 3467–3636 mL/kg/hr at 3 months, 3125–3203 mL/kg/hr at 6 months, and 2543–3022 mL/kg/hr at 9 months), and V_CO_ also differed overall for all levels of Pb exposure across the adult life-course in both females and males (p<0.0001 and p<0.0001, respectively). In females, V_CO_ was significantly higher relative to control for each of the three exposure groups at the 3-month time point (p = 0.03, p<0.01, p = 0.05 for 2.1 ppm, 16 ppm, and 32 ppm, respectively). By 6 months only the highest exposure level remained significant from control (p = 0.01). Interestingly, by 9 months, all three groups retained higher V_CO_, though the highest exposure group did not reach statistical significance (p = 0.03, p = 0.03, p = 0.08 for 2.1 ppm, 16 ppm, and 32 ppm, respectively). As with V_O_, in males the V_CO_ did not diverge from control until 9 months of age, when it was significantly reduced in the two higher exposure groups (p = 0.04, p = 0.02 for 16 ppm and 32 ppm, respectively) (Fig. 2a).

Respiratory exchange ratio (V_CO_/V_O_, RER) was calculated to identify the source of energy metabolism. By this measure, males showed a decrease in respiratory exchange, and only at 9 months for the two higher exposure groups (p = 0.02, p = 0.02 for 16 ppm and 32 ppm, respectively). Females did not show significant differences by exposure group for any time point.

### Food intake

Both sexes displayed increased average food intake over time with variable responses to Pb exposure. Overall food intake differed by exposure groups across the study for both females and males (p = 0.0008 and p<0.0001, respectively). Each exposure group was compared to the control group, and the linear trend was calculated across exposure groups at each time point (Fig. 2b). Females at the 32 ppm level showed significantly higher food intake than controls at the 9-month time point (2.61±0.09 g/day vs. 2.17±0.14 g/day) (p<0.01) with a significant increasing linear trend of across all exposures at the 9-month time point (p = 0.01). No groups showed changes in food intake at the two earlier time points, nor a significant linear trend. Males exhibited a similar pattern at the 6-month time point with a significant increase in food intake in the 32 ppm exposure group compared to control (2.71±0.04 g/day vs. 2.34±0.05 g/day) (p<0.01) and an increasing linear trend across all exposures (p<0.01). This effect plateaued by the 9-month time point, with the 16 ppm group eating significantly less food than control, and no linear trend was evident.

### Spontaneous activity

To further define the source of energy expenditure differences between groups, activity was measured in both horizontal and vertical movements and by ambulatory activity (Fig. S1). Overall horizontal activity was different across Pb exposures in females but not in males (p = 0.02 and p = 0.42, respectively). Female offspring exposed to 2.1 ppm Pb had higher average horizontal activity with 3082 counts/h at 9 months compared to 2626 counts/h in controls (p = 0.05) (Fig. S1a). At 3 months of age, females in the 32 ppm group decreased from an average of 2653 counts/h as compared to 3250 counts/h in control offspring (p = 0.02).

This sex-specific difference was mirrored in ambulatory measurement (subset of total horizontal activity) with only exposed females showing significant differences from controls (p = 0.01 and p = 0.59, females and males respectively) (Fig. S1b). Ambulatory activity was lower in females at the 32 ppm exposure level at 3 months with 1441 counts/h vs. 1856 counts/h in control offspring. Males did not exhibit significant differences at any time point or exposure level.

Although there was suggestive evidence of differences in the life-course patterns of vertical activity by exposure among females (p = 0.07) but not males (p = 0.35), exposed animals of neither sex showed significant differences at any specific time point as compared to control offspring (Fig. S1c).

### Body weight

**Figure 3:**
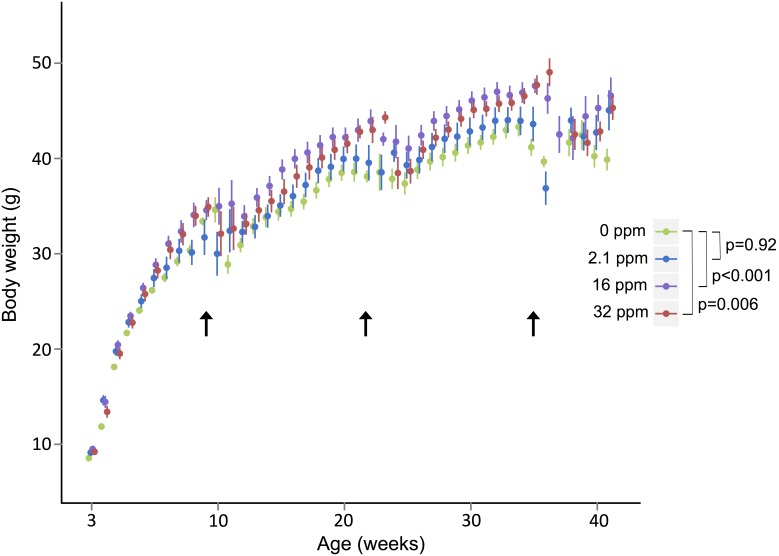
Weekly Body Weight Measurements. Male offspring across all exposure groups (grams) were weighed. Both medium and high exposure groups are significantly increased in weight across the life course as compared to control mice (in green). Arrows indicate weeks when mice were transferred for physiological parameter testing resulting in stress-induced weight loss. Bars represent 95% confidence intervals. P-values were obtained by adjusting for litter, and the autoregressive structure within mice, and adjusting for week using indicator variables for week of measurement.

When measured weekly at the colony housing location, male (p<0.0001) but not female (p = 0.77) mice showed sustained Pb-dependent differences in body weight. Compared to controls, males in the two highest exposure groups were heavier (p<0.001 and p = 0.006 for 16 ppm and 32 ppm, respectively) (Fig. 3). Three transport events to and from the physiological testing facility caused stress-induced fluctuations in weight across all groups, before a return to normal weight gain patterns as indicated by arrows in Figure 3. During the periods when the mice were at the colony housing location, 16 ppm exposed male mice were on average 3.4±1.4 g (p = 0.02) and 3.9±1.4 g (p = 0.01) heavier than control mice during weeks 12–22 and 24–35, respectively; 32 ppm exposed male mice were on average 2.8±1.4 g (p = 0.05) and 3.2±1.4 g (p = 0.02) heavier than control mice during weeks 12–22 and 24–35, respectively. Given the average weight of control male mice during those time periods (35.2±1.7 and 40.1±1.2 g, respectively), these exposure-related differences represent approximately an 8 to 10% increase in body weight. Weights at MNORC time points are given in Table 1.

**Table 1:** Body Weights at 3, 6, and 9 Months.

	Body Weights								
			3 month		6 month		9 month		
Sex	Exposure	N	mean	se	mean	se	mean	se	
Male	0 ppm	18	30.35	0.61	36.34	0.73	40.91	0.64	
	2.1 ppm	16	29.47	0.85	37.90	1.30	42.12	1.31	
	16 ppm	18	31.46	0.69	39.51	0.88	44.26	1.08	
	32 ppm	17	32.54	0.80	39.53	1.07	43.45	0.97	
Female	0 ppm	12	23.38	0.92	28.44	1.35	32.82	1.89	
	2.1 ppm	12	22.30	0.64	27.46	1.08	32.28	1.30	
	16 ppm	15	22.53	0.41	27.56	0.74	31.26	0.95	
	32 ppm	12	23.59	0.59	28.70	0.85	31.92	1.01	

### Body composition

**Figure 4:**
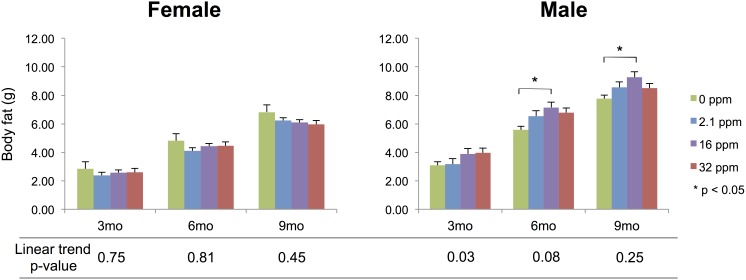
Body Fat. Measured by nuclear magnetic imaging. Females show no linear trend with exposure level. Males show a significant trend of increasing body fat with dose at 3 months, and near significance at 6 months. Stars indicate p-values<0.05 as compared to control offspring.

Overall, body fat differed heterogeneously among all Pb exposures for both females and males (p<0.0001 and p<0.0001, respectively) (Fig. 4). In males, a linear trend of increasing body fat across exposures was significant at 3 months (p = 0.03), but not significant at the 6-month (p = 0.08) and 9-month (p = 0.25) time points. No significant linear trend across exposures was seen in females at any time point, nor was an exposure level response observed when compared to controls at any time point. Comparing each exposure group to the control reveals a male-specific increase in body fat at the 6-month time point in the medium group (p = 0.02), and a significant increase at the 16 ppm exposure level at the 9-month time point (p = 0.03) (Fig. 4).

### Glucose tolerance test

**Figure 5:**
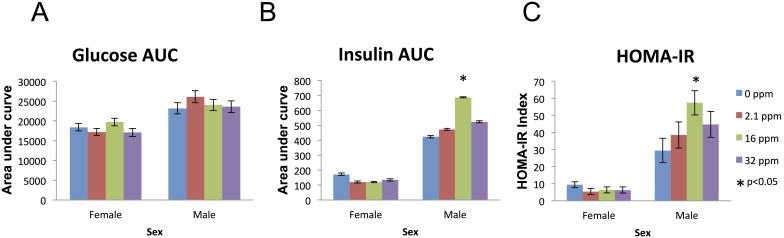
Blood Insulin and Glucose (9 months). (A) Glucose (mg/dl) measured by a fasting oral glucose tolerance AUC did not differ in either males or females. (B) Insulin (ng/ml) is increased in males at the medium exposure area under the curve (AUC) and (C) Homeostatic Model Assessment of Insulin Resistance (HOMA-IR). Calculated from baseline blood insulin and glucose levels, indicates an index of insulin resistance. Males at the medium exposure level show a significant increase in HOMA-IR as over control. Single stars indicate p-values<0.05, when compared to controls. Bars represent standard error.

Overall, baseline blood glucose did not differ between exposure groups in females (Fig. 5a). At a single time point (t = 15 min), 16 ppm exposed females had significantly increased blood glucose compared to controls (p = 0.01), but not at any other time point (Fig. S2a). Females also had no significant differences in insulin overall or at any time point (Fig. 5b). Overall, among males, there were no significant differences in blood glucose at baseline (Fig. 5a). At a single time point (t = 120 min), 2.1 ppm exposed male mice had a significantly increased blood glucose (p = 0.05) (Fig. S2a). However overall male insulin levels did differ, with 16 ppm exposed males having significantly increased insulin levels (p = 0.01), compared to controls (Fig. 5b). Furthermore, at each time point they maintained increased insulin levels compared to controls at t = 15 min (p = 0.01), t = 30 min (p = 0.03), t = 60 min (p = 0.05), and t = 120 min (p = 0.03), with a significantly increased area under the curve (AUC p = 0.02), indicating greater total insulin production throughout the test, compared to controls (Figs. 5b & S2b). The homeostasis model assessment-estimated insulin resistance (HOMA-IR) was significantly increased in 16 ppm exposed males (p = 0.01) compared to controls. There were no differences in HOMA-IR among females (Fig. 5c).

## Discussion

Early life exposures may have long-term consequences on the health of mammals, as outlined by the developmental origins of health and disease (DOHaD) hypothesis [1]. In this study, offspring exposed to Pb during development and lactation incurred persistent life-course changes in energy expenditure, food intake, body weight, total body fat, glucose tolerance and insulin response.

Several other exposure studies have suggested that metabolic phenotyping, including body composition, spontaneous motor activity, and energy expenditure, provides greater insight into the full phenotypic outcome of exposure than a simple measurement of body weight [26], [30], [31]. In our study, we consistently find sex-specific differences in the offspring by several measures. At 3 months of age, Pb-exposed females show energy expenditure changes, but by 6 months of age this effect dissipates for all but the highest exposed group, and by 9 months no effect remains. In contrast, exposed males do not differ from controls early in life, but show dose-dependent respiratory exchange rates later in life. The respiratory exchange ratio reflects these findings, with male offspring displaying significant differences at 9 months of age. As seen in previous reports, the RER in the control group showed a natural decline with age [26], [32]. Sex-specific differences are seen in aspects as diverse as earlier onset to puberty in female mice at low doses (2–13 µg/dL) to increases in human blood pressure in girls concomitant with perinatal Pb-exposure [33], [34]. Limited studies have examined Pb’s direct effects on energy metabolism. Struzyńska et al. report increased oxygen consumption Pb-exposed in rat neuronal synaptosomes [35], and Pb exposure is known to increase oxidative stress leading to multiple deleterious effects [36].

Feeding behavior may be influenced by Pb exposure level, sex, and species. Food intake decreases in rats chronically exposed to high levels of Pb-acetate (500 ppm) and was associated with depressive symptoms [37]. In contrast, our study found that food intake in female offspring is increased with 32 ppm Pb exposure levels at 9 months of age. In males this trend appears by 6 months but plateaus by 9 months. We interpret the earlier onset of male overeating as a more rapid response by males to their early life exposure, with females recapitulating this behavior in a delayed fashion. Contrarily, early life over-nutrition in small litters of mice has been found to increase wean weight and decrease physical activity, while having no influence on later life food intake, suggesting a decoupling of these metabolic processes as a result of changes in the perinatal environment [31]. One limitation of our study, however, is the potential for changes in dam’s water and food intake during the exposure due to the taste of Pb-acetate treated water. We chose to forgo dam water and food intake measurements in gestation in order to reduce potential confounding by maternal stress. Additionally, during late weaning, offspring pups may be directly exposed to Pb through direct water consumption.

By tracking weekly weights, we observed a similar sex-specific response that matches expectations from the food intake measurements. Previously, in this cohort of mice, we found that Pb exposure resulted in a trend of increased weight at weaning in males only; however, it was unknown whether the discordance in weight would remain as a life-long effect after the cessation of Pb exposure [29]. By three months of age, the male body weight differences are visually apparent (Fig. 3) and only slightly diverge in magnitude by the end of the study. Unlike males, the females did not exhibit an exposure-dependent increase in weight at weaning or across the life-course. Similarly, Leasure et al. found an increase in weaning weight for male mice exposed to low levels of Pb, though their results did not reach statistical significance until the 1 year time point [9]. We find that early life exposure results in permanent increases in body weight in an exposure-dependent manner, up to 9 months after the animals were challenged. Male mice exposed to medium, and high doses of Pb during gestation and lactation developed and maintained higher body weight throughout the life-course compared to unexposed mice. Male activity did not decrease significantly, however, by 9 months all Pb exposed groups showed reduced X-activity and X-ambulatory activity (representing walking), consistent with Leasure et al. who showed 1-year-old gestationally exposed mice male mice exhibited reduced cumulative activity [9].

Females in higher doses have increased food intake yet show no corresponding increase in weight, which may be a consequence of the greater time delay before onset of significant differences in food intake or the 9 month increase in spontaneous activity that was restricted to females. Human studies show conflicting evidence with Pb measured in teeth positively correlating with BMI at age 7, while Pb levels in patella and tibia of children the same age did not correlate with BMI, and cross-sectional studies of blood lead level found no association with obesity in 11 year old children or adult women [10], [38]–[40]. In contrast, Pb had a negative association with BMI and waist circumference in the NHANES 1999–2000 cohort [11]. The increase in body weight with exposure level may be a consequence of this energy/calorie imbalance. Male mice can maintain elevated body weight and fat even after the removal of obesogenic diets, suggesting small early shifts in metabolism can have long term effects at the organismal level and potentially on the microbiome [41]. Other causes such as elevated irisin levels have also been shown to increase energy expenditure with no changes in food intake or movement [42].

In mice fed high-fat diets for 7 weeks before being switched to control chow, the high fat groups maintained obese phenotypes yet serum insulin and glucose returned to control levels. In contrast to our results with Pb where serum insulin remained high in the medium exposure males [41]. Therefore toxicant early exposures can induce irreversible changes, unlike those induced by early high-fat diet exposure. The Pb-exposed male offspring display increased blood insulin levels following an oral glucose tolerance test with blood glucose levels remaining unchanged at 9 months of age. The HOMA-IR calculation indicates insulin resistance among the Pb-exposed males signifying an abnormal response to insulin in order to properly transport glucose. The insulin resistant response is likely a consequence of inefficient glucose cellular uptake from the increased body weight and fat observed throughout the male offspring’s life-course.

Due to the low Pb exposures used, and the extended period between perinatal exposures and adult measurements, we expected relatively small effect sizes. A longer exposure period and/or a longer measurement time course may reveal the effects of increased food intake on female body weight, among other metabolic phenotypes that become more dramatic towards senescence. Further, increased power would enhance the ability to detect interactions between the metabolic endpoints. Our goal in this study design was to evaluate the effect of early life exposures on adult animals, prior to age-related changes in metabolic homeostasis. Given the significant findings shown here, however, we argue that follow-up studies should also incorporate geriatric time points.

The mechanism underlying the persistent effects of early life Pb exposure remains unresolved, however evidence suggests that epigenetic programming factors are likely responsible [43]. Recent reports find sex-specific effects on Dnmt1, Dnmt3a, and MeCP2 expression in rat hippocampi even at low doses and that Pb-exposure dependent epigenetic effects are most sensitive during the early developmental window [44]. We recently found shifts in transposon-associated gene methylation and body weight at weaning after early life Pb exposure, yet it was unknown if physiological differences would be maintained throughout the life course, as we have shown here [29].

## Conclusions

Mice exposed to lead (Pb) from conception to weaning showed in adulthood increases in food intake, reflected in increased body weight, and changes in energy expenditure, activity, glucose tolerance, and insulin response. These physiological effects associated with obesity persist into adulthood and vary according to sex and age.

## Supporting Information

Figure S1 **Spontaneous Activity. Measured in counts/h.** (A) Females show a significant decrease in Horizontal Activity at 3 months (32 ppm) and increase at 9 months (2.1 ppm). (B) Females show a significant decrease in Ambulatory Activity at 3 months (32 ppm). (C) Vertical Activity exhibited no change in either sex at any time point or exposure level.
(TIFF)

Figure S2 **Blood Glucose and Insulin over Time Course (9 months).** (A) Glucose (mg/dl) measured by a fasting oral glucose tolerance test at 0 (baseline), 15, 30, 60, and 120 minutes shows an increase only at the medium exposure level in females and low exposure level in males at single time points. (B) Insulin (ng/ml) is increased in males across all time points at the medium exposure and Single stars indicate p-values<0.05, double stars indicate p-values<0.01 when compared to controls.
(TIFF)

File S1 **Physiological measurement statistics.** Means and standard deviations for physiological parameters by sex, exposure, and measurement time point.
(XLS)

Methods S1 **Measurements for animal exposure, life course evaluation, Pb measurements, and statistical software.**
(DOCX)
