# Supplementary figures and images for: Perinatal Lead (Pb) Exposure Results in Sex-Specific Effects on Food Intake, Fat, Weight, and Insulin Response across the Murine Life-Course
Source: PLoS One. 2014 Aug 8;9(8):e104273. doi: 10.1371/journal.pone.0104273 (PMC4126699; doi:10.1371/journal.pone.0104273)

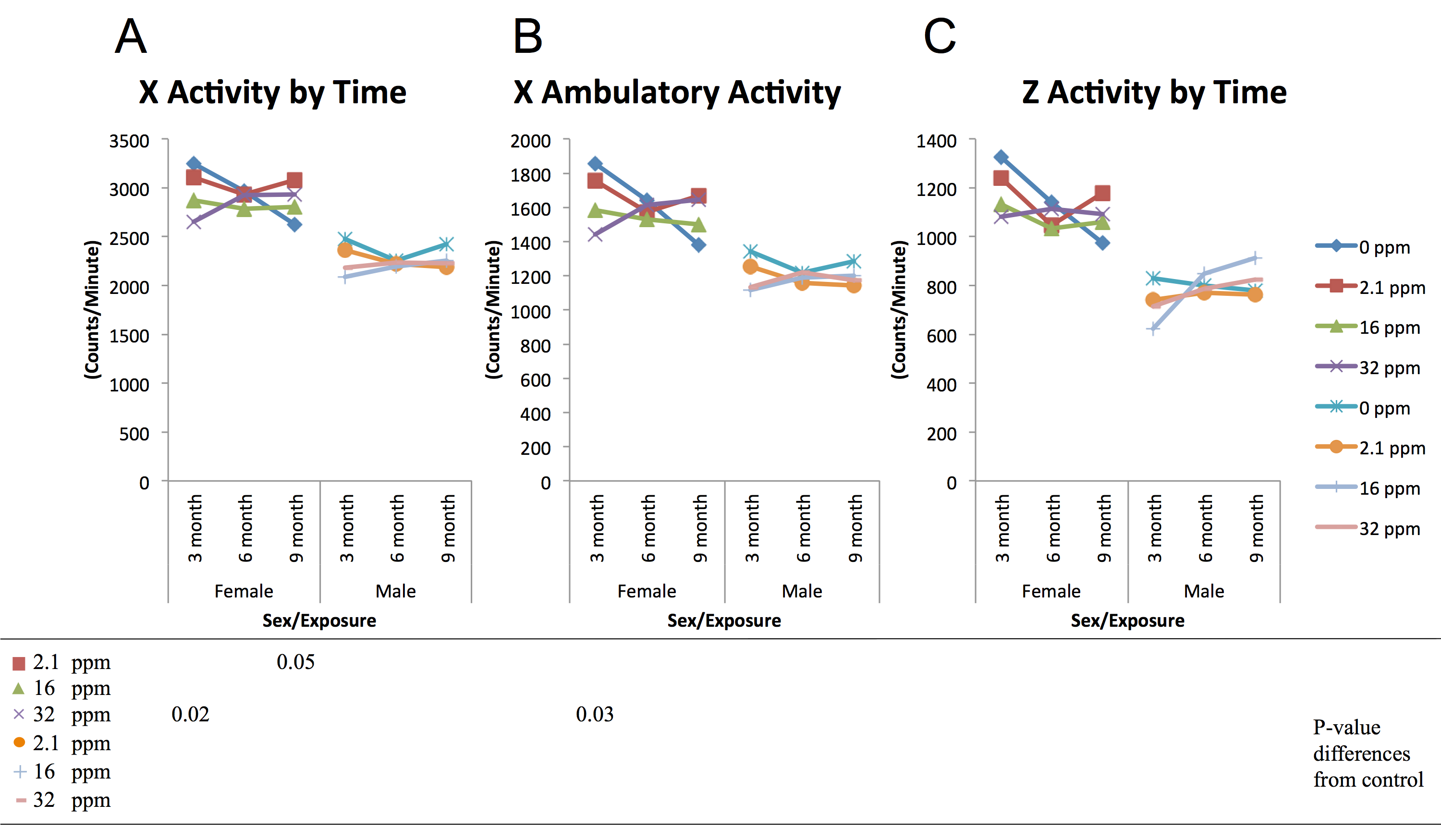

Supplement: Figure S1 — Spontaneous Activity. Measured in counts/h. (A) Females show a significant decrease in Horizontal Activity at 3 months (32 ppm) and increase at 9 months (2.1 ppm). (B) Females show a significant decrease in Ambulatory Activity at 3 months (32 ppm). (C) Vertical Activity exhibited no change in either sex at any time point or exposure level. (TIFF) [file pone.0104273.s001.tiff]

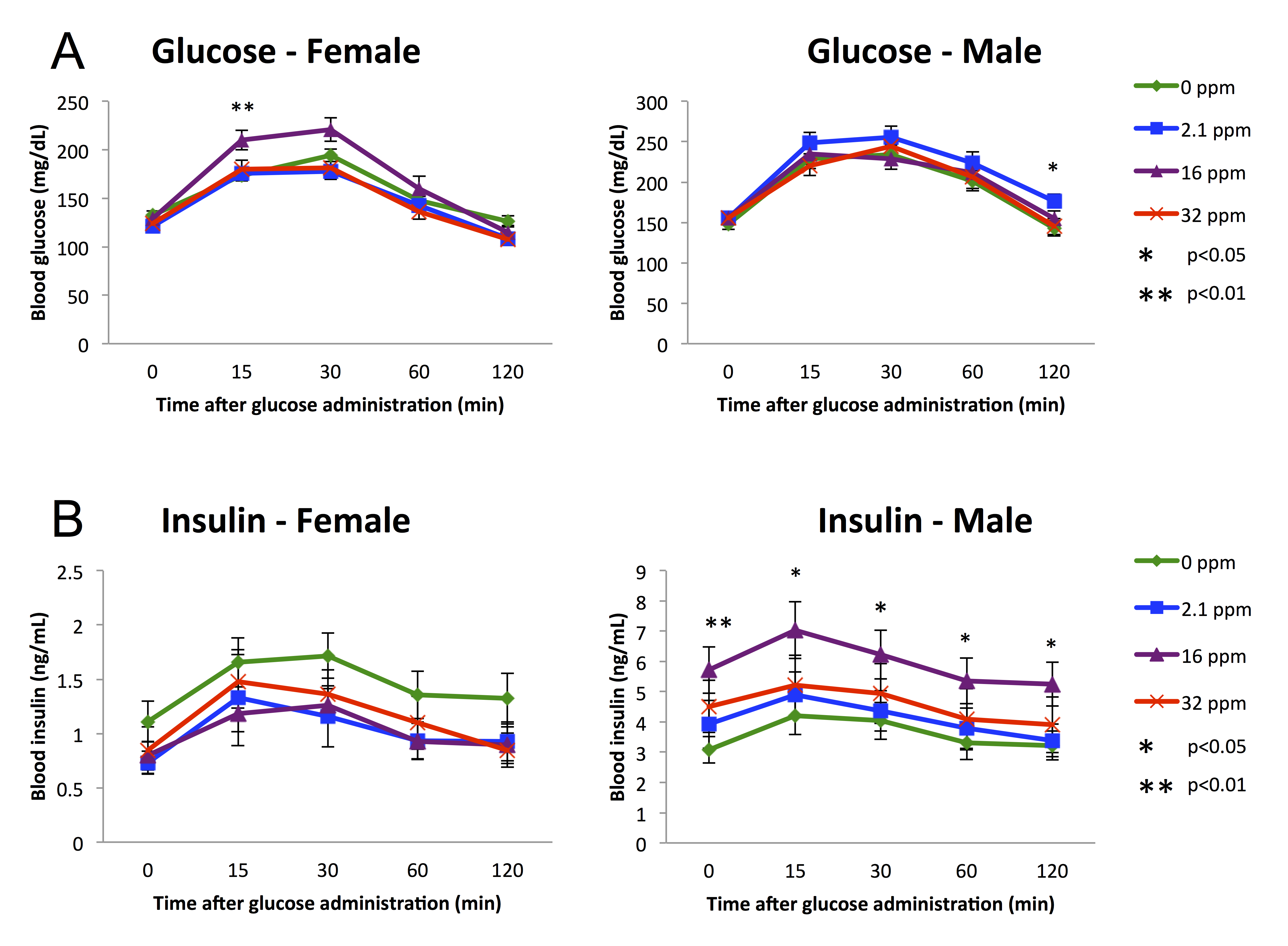

Supplement: Figure S2 — Blood Glucose and Insulin over Time Course (9 months). (A) Glucose (mg/dl) measured by a fasting oral glucose tolerance test at 0 (baseline), 15, 30, 60, and 120 minutes shows an increase only at the medium exposure level in females and low exposure level in males at single time points. (B) Insulin (ng/ml) is increased in males across all time points at the medium exposure and Single stars indicate p-values<0.05, double stars indicate p-values<0.01 when compared to controls. (TIFF) [file pone.0104273.s002.tiff]
