# Supplementary material for: Perinatal Lead (Pb) Exposure Results in Sex-Specific Effects on Food Intake, Fat, Weight, and Insulin Response across the Murine Life-Course
Source: PLoS One. 2014 Aug 8;9(8):e104273. doi: 10.1371/journal.pone.0104273 (PMC4126699; doi:10.1371/journal.pone.0104273)
Supplement: Methods S1 — Measurements for animal exposure, life course evaluation, Pb measurements, and statistical software. (DOCX) [file pone.0104273.s004.docx]

**Perinatal Lead (Pb) Exposure Results in Sex-Specific Effects on Food Intake, Fat, Weight, and Insulin Response across the Murine Life-Course**

Christopher Faulk^1‡^, Amanda Barks^1‡^, Brisa N. Sánchez^2^, Zhenzhen Zhang^2^, Olivia S. Anderson^1^, Karen E. Peterson^1, 3, 4^, and Dana C. Dolinoy^1§^

**Supplementary Material**

Supplementary Methods S1.

*Animals and exposure*

Animals were acclimated for 7 days on a 12-hour light-dark cycle. Following acclimation, animals were placed in an open-circuit indirect calorimeter [Comprehensive Lab Animal Monitoring System (CLAMS), Columbus Instruments, Columbus, OH, USA] for 72 hours, comprised of 12-hour light-dark cycles, with direct access to food and water. The CLAMS chamber measured energy expenditure by sampling the chamber gas composition sequentially for 5 seconds in 10 minute intervals, resulting in measures of oxygen consumption (V_O_), carbon dioxide production (V_CO_), and respiratory exchange ratio (RER = V_CO_ / V_O_). To measure spontaneous motor activity, the chamber was equipped with an optical beam activity-monitoring device in both the horizontal and vertical directions to capture ambulatory movement when two IR beams are broken, indicating movement over space (walking), total horizontal (ambulatory plus additional horizontal movement such as change in posture or turning activity, when only a single IR beam is broken), and vertical dimensions. Each interruption of a beam by animal movement was recorded as a “count” with measurements in units of counts/hour. Finally, the CLAMS chamber was equipped with a feeding device and scale that continuously measured the amount of powdered food eaten in each 12-hour interval in 20-minute increments, and washed with 10% bleach solution between each animal measurement. Body composition, including body fat, lean mass, and free fluid, were measured with an nuclear magnetic resonance (NMR) analyzer (Minispec LF90II, Bruker Optics) following the 72-hour CLAMS monitoring. Weight was taken immediately before placing animals in the CLAMS chamber. In addition to the 3, 6, and 9-month time points, *a/a* mice were weighed on a weekly basis, starting at weaning. Weight measurements were not taken during weeks when animals were in transfer to or from the MNORC. Glucose tolerance tests were performed on all animals after the 9 month time point using tail vein puncture following a 5 hour fast.

*Life course evaluation*

Blood samples were collected immediately prior to and after the gavage at 0 (baseline), 15, 30, 60, and 120 minutes for measurement of glucose and insulin. Blood glucose was measured with a glucometer (Acucheck, Roche). Serum insulin levels were measured by enzyme-linked immunosorbent assay (ELISA; Millipore, St. Charles, MO).

*Pb Measurements*

We calculated theoretical values for Pb-acetate dissolved in water to coincide with final concentrations of 0 ppm, 3.7 ppm (9.75x10^-6^ M), 27 ppm (7.12x10^-5^ M), and 55 ppm (1.45x10^-4^ M). Upon testing of the resulting solutions via ICPMS, we found actual concentrations of 0 ppm, 2.1 ppm (5.54x10^-6^ M), 16 ppm (4.22x10^-5^ M), and 32 ppm (8.44x10^-5^ M).

Our analysis of the Pb content of treated water and the resulting maternal blood lead suggests variability between resulting blood lead levels (BLL) found in previous studies may not directly correlate to theoretical exposure levels. For example, Leasure et al. exposed mice to 55 ppm Pb-acetate in drinking water during gestation and measured blood lead level at 24-27 μg/dL in trunk blood at time of weaning (similar to our results) while Mesdaghinia et al. exposed 2-3 month old male mice to 50 ppm Pb-acetate in drinking water for 30 days and measured only 6.35 μg/dL lead in heart blood ([Leasure et al. 2008a](#_ENREF_7); [Mesdaghinia et al. 2010](#_ENREF_45)). Our maternal BLL were calculated from heart blood, collected by cardiac puncture. Relatively low levels of exposure, here represented by our 2.1 ppm treatment group, result in human relevant blood lead levels, 4.1 (±1.3) μg/dL, which is near the current CDC recommended action level guidelines of 5 μg/dL ([Betts 2012](#_ENREF_46)). It is important to note that blood Pb levels will respond to physiological conditions such as pregnancy, influencing blood, organ, and tissue Pb levels as well as being affected in the dam during gestation and lactation, therefore direct comparisons to other studies should be avoided.

*Statistical software*

Statistical analyses were carried out using SAS 9.3 (SAS Institutes, Cary, NC) and R 2.13.2 (<http://www.r-project.org/>) using the lme4 package for liner mixed models. P-values for linear trend analyses were calculated using Markov chain Monte Carlo resampling (pvals.func). Results were considered significant at values of p ≤ 0.05 and marginally significant at values of p> 0.05 and ≤0.10.
